# Does tourniquet use affect the periprosthetic bone cement penetration in total knee arthroplasty? A meta-analysis

**DOI:** 10.1186/s13018-020-02106-6

**Published:** 2020-12-11

**Authors:** Chao Lu, Min Song, Jin Chen, Congcong Li, Wenzheng Lin, Guozhu Ye, Gaoyi Wu, Anan Li, Yuning Cai, Huai Wu, Wengang Liu, Xuemeng Xu

**Affiliations:** 1 Orthopedics Department, Guangdong Second Traditional Chinese Medicine Hospital, NO.60 Hengfu road, Guangzhou, 510095 Guangdong China; 2 Fifth Clinical Medical School, Guangzhou University of Chinese Medicine, Guangzhou, 510095 Guangdong China

**Keywords:** Tourniquet, Total knee arthroplasty, Bone cement, Postoperative pain, Meta-analysis

## Abstract

**Background:**

A tourniquet is a device commonly used to control massive hemorrhage during knee replacement surgery. However, the question remains whether the use of tourniquets affects the permeability of the bone cement around the knee prosthesis. Moreover, the long-term effects and stability of the knee prosthesis are still debatable. The aim of this study was to examine whether the use of a tourniquet increases the thickness of the cement mantle and affects the postoperative blood loss and pain during primary total knee arthroplasty (TKA) using meta-analysis.

**Methods:**

We searched the Cochrane Central Library, MEDLINE, Embase, PubMed, CNKI, and Wang Fang databases for randomized controlled trials (RCTs) on primary TKA, from inception to November 2019. All RCTs in primary TKA with and without a tourniquet were included. The meta-analysis was conducted using RevMan 5.2 software.

**Results:**

A total of eight RCTs (677 knees) were analyzed. We found no significant difference in the age and sex of the patients. The results showed that the application of tourniquet affects the thickness of the bone cement around the tibial prosthesis (WMD = 0.16, 95%CI = 0.11 to 0.21, *p* < 0.00001). However, in our study, there was no significant difference in postoperative blood loss between the two groups was observed (WMD = 12.07, 95%CI = − 78.63 to 102.77, *p* = 0.79). The use of an intraoperative tourniquet can increase the intensity of postoperative pain (WMD = 1.34, 95%, CI = 0.32 to 2.36, *p* = 0.01).

**Conclusions:**

Tourniquet application increases the thickness of the bone cement around the prosthesis and may thus increase the stability and durability of the prosthesis after TKA. The application of an intraoperative tourniquet can increase the intensity of postoperative pain.

## Background

Total knee arthroplasty (TKA) is a common and effective treatment method for severe knee arthritis. Previous studies have shown that the patient satisfaction rate after TKA is up to 91% [1]. However, one of the most important problems encountered by patients is the high rate of revision TKA for the loosening of the tibial component [2]. There are many reasons for the aseptic loosening of the prosthesis, such as infection, wear and tear, unbalance of force line, and loosening of the bone-cement interface [3]. The thickness of the bone cement is critical for the success of primary TKA [4, 5]. Factors such as bone preparation, use of cement gun or artificial filling, and bone cement penetration during TKAs are related to the thickness of bone cement around the prosthesis [6, 7]. Increased thickness of the bone cement has been justified to improve prosthesis survival and stability [8–11]. Recent studies have found that a revision rate of 2–5% is due to the loosening of the prosthesis [12]. The cement covering 3–4 mm between the tibial implant and the tibial trabecular bone is considered to be the best option to avoid osteolysis and loosening of the surrounding bone [3]. The use of tourniquets during TKA is highly recommended by the surgeon. Some studies have found that the use of tourniquets causes an increase in cement penetration due to loss of the blood and fat in the cancellous bone during bonding [13, 14]. However, there are also studies that suggest that tourniquet use does not affect cement mantle penetration [15–18], further clouding the issue. It is unclear whether the use of tourniquets can increase the thickness of the bone cement around the tibial prosthesis and indirectly improve the stability and durability of the prosthesis. Therefore, we conducted a meta-analysis of randomized controlled trials (RCTs) to determine whether the use of tourniquets affected the thickness of the bone cement around the prosthesis.

## Materials and methods

### Methodology

The meta-analysis was carried out in accordance with the Preferred Reporting Items for Systematic Reviews and the Guidelines for Meta-Analysis (PRISMA) [19]. Biomedical databases such as PubMed, the Cochrane Library, Embase, MEDLINE, CNKI, and Wanfang were extensively searched for clinical studies with no language constraints evaluating the use of a tourniquet or not in TKA, from inception to November 2019. RevMan 5.2 software was used to carry out the meta-analysis. The following keywords were used to optimize search: tourniquet and bone cement and (total knee arthroplasty or arthroplasties, replacement, knee or arthroplasty, knee replacement or knee replacement arthroplasties or replacement arthroplasties or knee replacement arthroplasty or knee prosthesis). In addition, the reference lists of the reported papers were checked to look for extra studies that might meet the research requirements.

### Criteria for inclusion and exclusion

For this meta-analysis, we established the following inclusion criteria: (1) patients who received primary TKA, (2) RCTs, (3) comparing the outcomes with or without a tourniquet in TKA, and (4) outcome measurements should include these parameters (thickness of the bone cement, VAS knee pain scores, calculated blood loss, and complications).

Exclusion criteria were (1) non-RCTs, (2) unpublished results, (3) meeting proceedings, and (4) TKA revision.

### Data extraction

Data were collected by two researchers separately in the same layout and after verified by a third party, and all disagreements were resolved by a consensus. We approached the authors for missing data and supporting information wherever necessary. Data related to the publication, demographics, time of tourniquet, time of the procedure, bone cement thickness, blood loss rates, VAS knee pain ratings, and complications (minor and major) were all included.

A complication can be minor or major depending on the need for a second operation. We have identified major complications such as wound dehiscence, vessel injuries, active hemorrhage, infections and hematomas requiring drainage or debriding or revision, and extreme knee rigidity requiring anesthesia manipulation. We identified minor complications such as wound complications such as deep vein thrombosis (DVT), marginal necrosis, oozing, mild knee stiffness, erythema, superficial infection, and severe leg swelling that could be treated by conventional therapy and do not require any further surgery.

### Quality assessment

Two investigators separately evaluated the likelihood of bias in the included studies using the following items: blinding of subjects, randomization, incomplete results, allocation concealment, blinding of the assessment of outcomes, selective reporting of results, and other biases [20]. The item was reported as “small,” “high,” or “uncertain” based on the information provided by the included studies. Small indicates low-bias risk, high indicates high-bias risk, and unclear indicates a lack of information or unspecified bias risk.

### Statistical analysis

Meta-analysis was performed using RevMan 5.2 software (Cochrane Collaboration, Oxford, UK). The odds ratio (OR) with 95% confidence intervals (CIs) was determined for dichotomous outcomes, and the mean difference (MD) with 95%CIs was used for continuous outcomes. A *p* value less than 0.05 was considered statistically significant (*p* < 0.05). Heterogeneities in the studies were examined using the *I*^2^ statistic, and we defined the substantial heterogeneity as *I*^2^ value greater than 50%. If the meta-analysis showed a substantial heterogeneity, we used a model of random effect; otherwise, we used a model of fixed-effect.

## Results

**Fig. 1:**
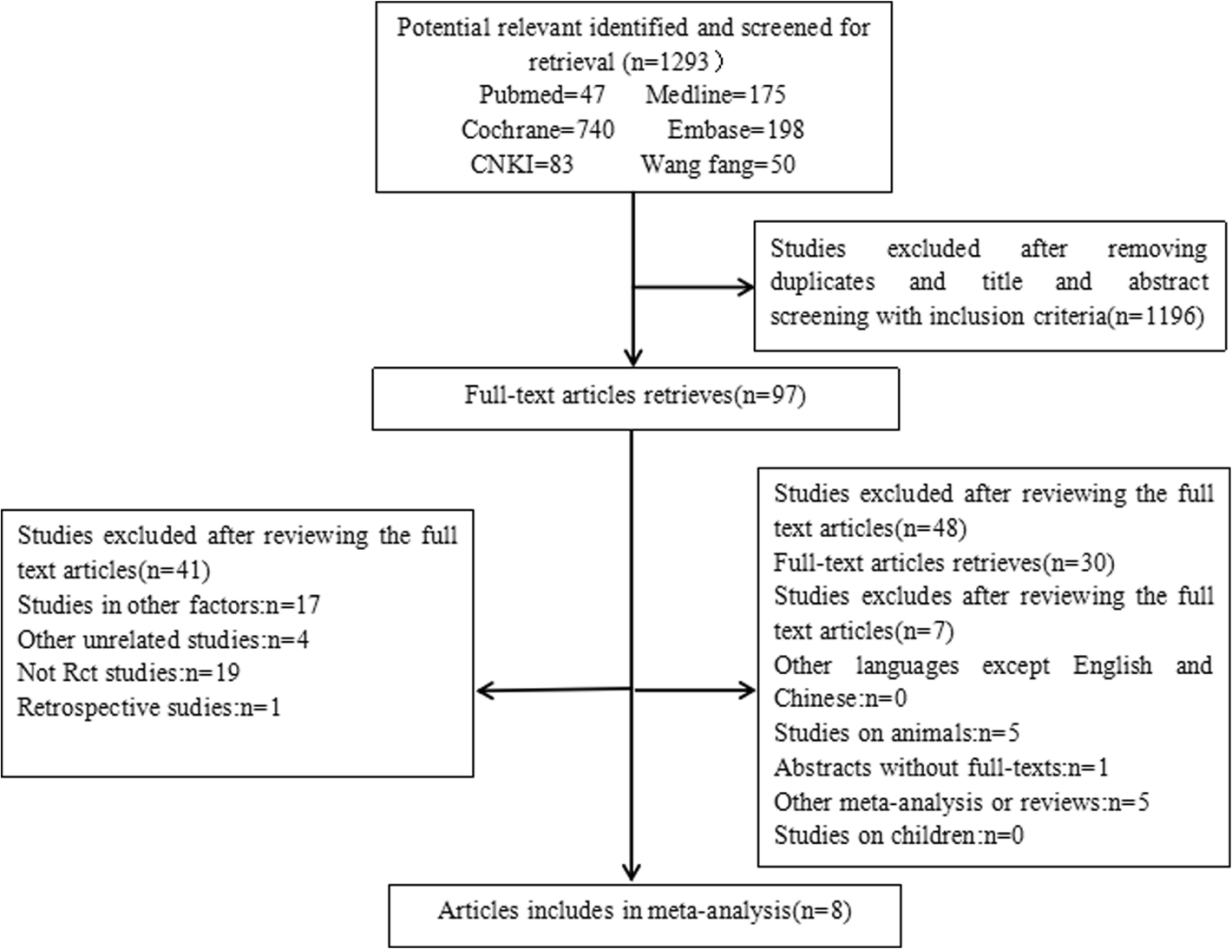
The flow chart of literature screening

After a thorough evaluation, in the overall meta-analysis, eight separate RCTs with a combined sample size of the 677 knees (Fig. 1).

### Risk of bias assessment

**Fig. 2:**
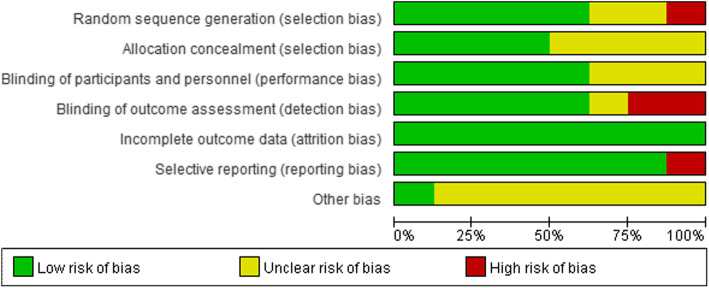
Risk of bias methodological quality

**Fig. 3:**
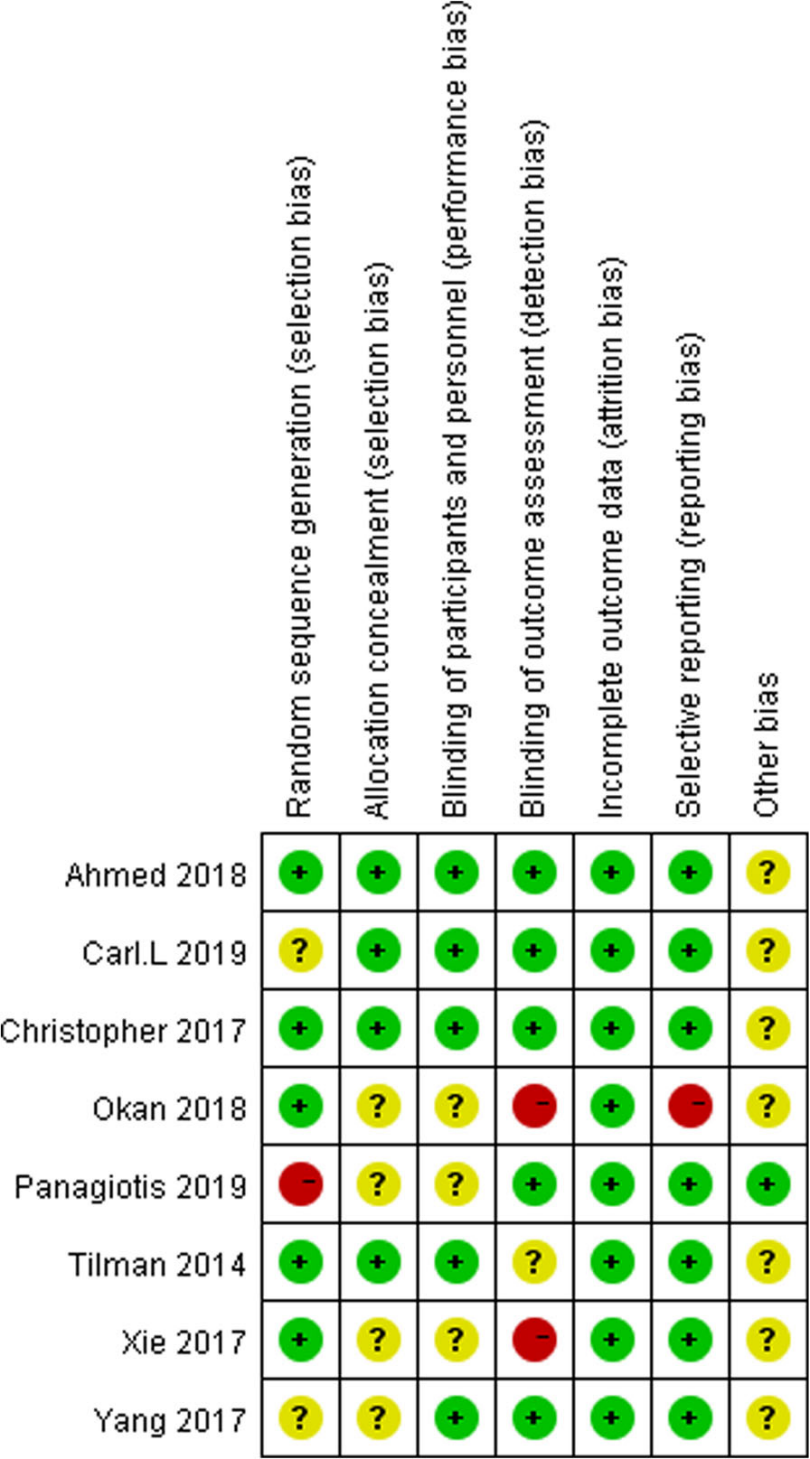
Risk of bias summary

Most of the studies were relatively well designed. However, several studies did not provide a specific description of the randomization method, which could result in selection bias. Many studies have been short-term, making long-term assessment indexes scarce. Out of the eight selected studies, only in one study, the follow-up duration was 3 years. Another study was followed-up for 1 month after surgery. The rest only observed changes in patient indicators within 1 week after surgery. The methodology quality and the summary of the risk of bias are shown in Figs. 2 and 3, respectively.

### Outcome measure

#### Bone cement penetration

**Fig. 4:**
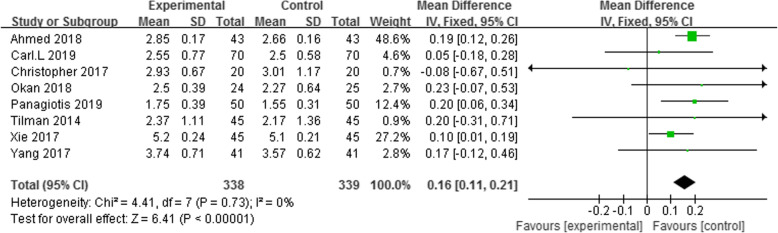
Forest plot of cement penetration depth

Eight studies reported on bone cement penetration [13–18, 21, 22]. Anterior-posterior (AP) and lateral (Lat) view X-rays to measure the cement mantle thickness under the tibial component. The results suggest that the application of tourniquets can increase the thickness of the bone cement around the tibial prosthesis (WMD = 0.16, 95% CI = (0.11 to 0.21), *p* < 0.00001, *I*^2^ = 0%) (Fig. 4).

#### Pain assessment

**Fig. 5:**
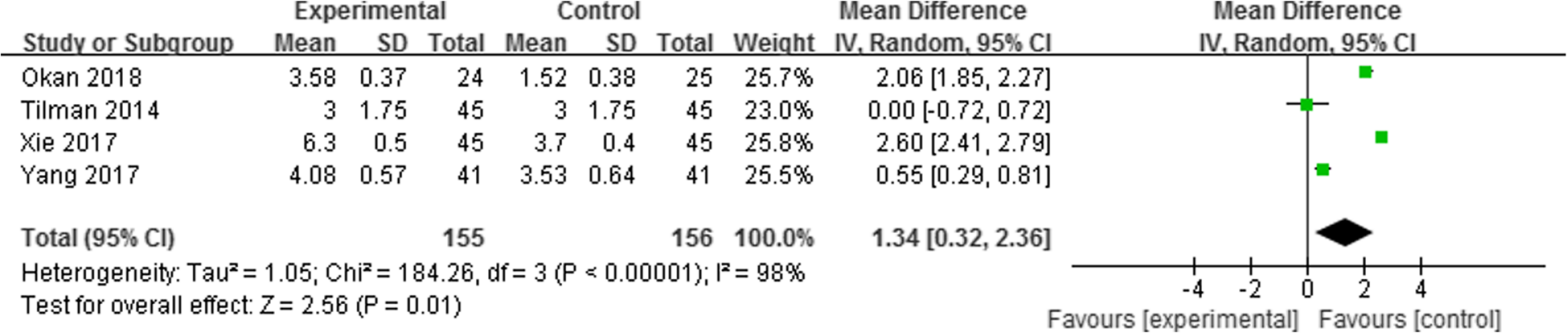
Forest plot of VAS knee pain scores

Four studies reported on the VAS knee pain scores [14, 18, 21, 22]. Pooled results showed that tourniquet use increased postoperative pain (WMD = 1.34, 95% CI = (0.32 to 2.36), *p* = 0.01, *I*^2^ = 98%) (Fig. 5).

#### Blood loss

**Fig. 6:**
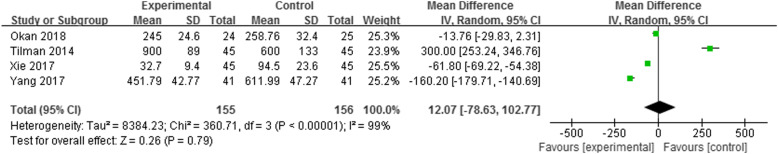
Forest plot of intraoperative blood loss

Four studies provided data on the calculated blood loss [14, 18, 21, 22]. We found no major difference in calculated blood loss between the four groups (WMD = 12.07, 95%CI = (− 78.63 to 102.77); *p* = 0.79, *I*^2^ = 99%) (Fig. 6). The reason for this result may be because blood loss calculation methods in the different literature are not uniform and the application schemes and the inflation time of tourniquets are not consistent. The lack of data may be the contributing factor.

#### Complications

Complications were reported in three studies [15, 17, 22]. The number of patients with minor complications increased following tourniquet use. There was no reference to pulmonary embolism. In the tourniquet group, DVT occurred in 22 patients, and the surgical site was infected in 1 patient, but revision surgery was not required. One patient was diagnosed with high skin temperature of joints, and 31 patients were diagnosed with subcutaneous ecchymosis. One patient had a sluggish wound healing in the non-tourniquet group with no need for reconstruction operation, three patients were diagnosed with subcutaneous ecchymosis, and 6 patients were diagnosed with DVT.

## Discussion

To the best of our knowledge, this study is the first meta-analysis of RCTs to compare the use of a tourniquet with the thickness of the cement mantle in TKA. A significant conclusion of this study was that the use of tourniquets will increase bone cement thickness. Moreover, the application of tourniquets can significantly increase postoperative pain. TKA is a common and successful procedure, with over 1 million TKAs performed annually in OECD countries [23]. According to a survey, tourniquets are commonly used in TKA operations between 37% and 93% [9, 24, 25]. Li et al. observed that tourniquet use will decrease intraoperative bleeding and increase the field of view in a recent meta-analysis [26]. However, tourniquets have caused problems such as leg pain, inflammation of the arms, nerve palsy, subcutaneous thigh fat necrosis, vascular damage, postoperative weakness, slow recovery of quadriceps power, complications of the wound, and DVT [27–32].

The finding shows that the use of tourniquets can significantly increase the thickness of the cement mantle. The use of an intraoperative tourniquet is one of the essential elements influencing the formation of TKA bone cement [14]. On the one hand, due to the bloodless field, the use of tourniquets offers a better vision which would contribute to improving cementing quality [33]. On the other hand, the formation of a bloodless surface and dry surface may affect the penetrating loosening of bone cement, which is mainly confined to bone cement structure [14]. Touzopoulos et al. [13, 14] observed that the use of tourniquets may decrease blood flow and fat in cancellous bone during cementing. The use of tourniquets can make the intraoperative trabecular shaft clean and bloodless, achieving the same effect as the suction technique, which has been shown to improve the cement thickness under the tibial component [34, 35].

Our results demonstrate that tourniquet application significantly increased postoperative pain. Many studies have shown that tourniquet use can increase postoperative pain in TKA patients [36, 37]. Studies have found that the extended use of tourniquets may further intensify hypoxia in the soft tissue around the cut, resulting in severe inflammation and muscle damage, which may enhance postoperative pain [35]. Also, the congestive swelling of the soft tissue capsule can cause greater invisible blood loss and direct damage to the nerve structure and soft tissue [36, 38–40]. Conversely, lower inflatable pressure and shorter tourniquet use can reduce postoperative pain and thus reduce complications [41, 42]. However, in our study, the time of pain measurement was not uniform. Two studies mentioned that pain was measured at 24 h and 48 h after operation, one study was measured after 4 days of surgery, and another was measured after 6 weeks of surgery. One study did not mention the specific time of measurement. All pain measurement was done at rest and during mobilization and documented on a VAS.

There was no substantial difference between the two groups in postoperative blood loss. The main reason for this finding may be that the methods used to calculate blood loss vary from study to study. Okan et al. calculated the blood transfusion volume and drainage volume 24 h after operation. Tilman et al. used Bourke and Smith’s method to calculate the total blood loss, taking into account both the dominant and the recessive blood loss. Xie et al. included the liquid in the suction bottle minus the liquid used intraoperatively plus the net weight of the gauze pad weight. Yang et al. measured negative pressure drainage bottle drainage volume within 24 h and intraoperative blood loss. The original purpose of using a tourniquet in TKA surgery was to reduce the blood loss. In theory, the premature release of a tourniquet results in a significant increase in both explicit and implicit blood loss. However, many studies have found that the effect of a tourniquet to reduce the blood loss is still controversial, and few researchers consider that tourniquet use may increase the blood loss. This may be due to increased “hidden blood loss” after the tourniquet use resulting in post-ischemic activated fibrinolysis [43, 44], such as hyperperfusion after deflation. Activation of the fibrinolytic enzyme can cause an increase in postoperative blood loss. In few studies, it was observed that the fibrinolytic activity maintained for a short time (30 min), which was not enough sufficient for the main cause of immense postoperative bleeding. The tourniquet was used in most of the studies; however, in our study, the pressure on the cuff inflated varies from 250 to 360 mmHg, and the statistical methods for the blood loss were also inconsistent, which may also be the reason for the non-significant difference between the two groups of patients.

Regarding postoperative complications, this meta-analysis suggests that the use of tourniquets increases the risk of complications. In our contained research, subcutaneous ecchymosis was the most widespread minor complications. The reason for that is because tourniquet use during surgery increases microvascular permeability leading to blood stagnation and hypercoagulation, which is prone to DVT [45, 46]. Certain studies AS reported that an extra 10 min in the operating theater was associated with an increase in complications [35]. Under the same surgical conditions, the use of a tourniquet may reduce the operation time and reduce the risk of infection to a certain extent, which may help to reduce the occurrence of potential complications.

There are some limitations to this study. First, the small sample size of the included studies, only eight RCTs have been included, which may have contributed to inexact findings and the complication percentages were not established. Second, the use of the tourniquet in the included studies was inconsistent; the pressure on the cuff inflated varies from 250 to 360 mmHg, which may have influenced the final outcome. Third, although some findings have been documented in the included studies, some details have not been adequately provided such as inflammation of the limbs, hemoglobin or hematocrit levels, deep vein thrombosis, tourniquet time, and joint function. In addition, only the cement mantle thickness of the tibial portion was examined. More assessment of the femoral mantle thickness and the use of three-dimensional computed tomography scans can provide a more accurate picture. Finally, in most RCTs, the follow-up time was comparatively short, which could neglect long-term effects such as the durability of prosthetic attachment and regeneration of joint function. A shorter and variable follow-up duration in most of the selected studies could be another limitation. Further prospective studies are needed in the future to confirm that tourniquet affects the stability and durability of the pretibial prosthesis of the TKA.

## Conclusions

In conclusion, the application of tourniquet in primary TKA increases the stiffness of the implant-cement-bone composite of the tibia and may have an impact on the long-term survival of the implant; however, its use also increases postoperative pain and its complications. Our study results will assist orthopedic surgeons when selecting the best surgical technique for their patients. In the future, further prospective studies are needed to evaluate the effect of tourniquet on the thickness of bone cement around the prosthesis. To validate this study, a large and well-designed RCT with comprehensive follow-up is required.

## Data Availability

The datasets used and analyzed during the current study are available from the corresponding author on reasonable request.

## Abbreviations

TKA: Total knee arthroplasty
RCTs: Randomized controlled trials
DVT: Deep vein thrombosis
